# Transfer Dynamics of Hydrophilic and Lipophilic Surfactants in Turbulent Oil–Water Emulsions

**DOI:** 10.1021/acs.langmuir.5c01250

**Published:** 2025-06-02

**Authors:** Umberto Baù, Francesca Mangani, Alessio Roccon, Alfredo Soldati

**Affiliations:** † Institute of Fluid Mechanics and Heat Transfer, TU-Wien, Vienna 1060, Austria; ‡ Department Engineering and Architecture, 9316 University of Udine, Udine 33100, Italy

## Abstract

We investigate the transfer dynamics of hydrophilic and lipophilic surfactants in turbulent oil-in-water emulsions, in which oil droplets are dispersed in a continuous water phase, with the surfactant hydrophilic–lipophilic balance (HLB) influencing its solubility in both phases. Using direct numerical simulations, we solve the turbulence dynamics in a closed channel, while the time evolution of the emulsion morphology and surfactant concentration field is obtained using a phase-field method (PFM) based on two Cahn–Hilliard-like equations obtained from a Ginzburg–Landau free energy functional. The effect of surfactant on interfacial tension is modeled via an equation of state. The PFM can account for varying solubility, distinguishing between water-soluble (high HLB), equally soluble (intermediate HLB), and oil-soluble (low HLB) surfactants. Our results show that while the overall topology of the dispersed phase remains relatively unaffected, significant differences arise in the surfactant distribution at the interface and in the bulk of the two phases. Oil-soluble surfactants exhibit higher concentrations at the interface compared to water-soluble ones. By analyzing surfactant concentration, transfer fluxes, and free energy, we identify the key mechanisms governing surfactant transport in turbulent emulsions.

## Graphical abstract

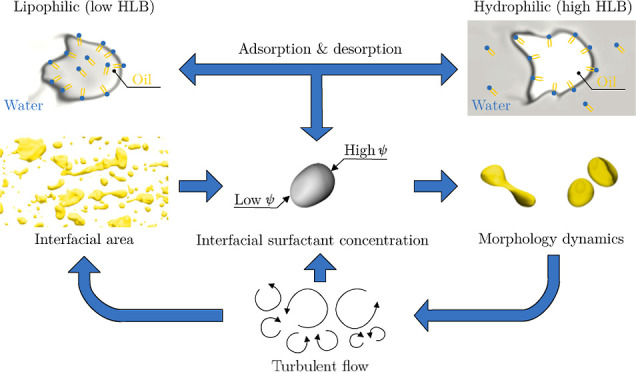

## Introduction

Surfactants are chemical compounds that modify interfacial properties by altering the balance of forces at the interface between two immiscible phases.^1,2^ Surfactant molecules consist of a hydrophilic head that interacts favorably with aqueous solvents and a hydrophobic tail that is attracted to organic solvents like oil. Due to their amphiphilic nature, surfactants tend to adsorb at the interface, orienting their heads toward the aqueous phase and their tails toward the organic phase, thereby minimizing the energetic cost of maintaining the interface. This adsorption reduces the local interfacial tension, a process that depends on the local concentration of surfactants. The concentration itself is influenced by the surfactants’ transport properties within the fluid as well as their solubility in the respective phases.^3,4^ In liquid–liquid systems, surfactants can exhibit selective solubility, dissolving either in both phases or predominantly in one.^2^ This solubility behavior is characterized by the hydrophilic–lipophilic balance (HLB),^5^ a numerical scale from 0 to 20 that quantifies a surfactant relative affinity for water (hydrophilic) or oil (lipophilic). Surfactants with a low HLB (e.g., Span-80) preferentially dissolve in the oil phase, while those with a high HLB (e.g., Tween-80) tend to dissolve in the water phase. This solubility difference influences the adsorption and desorption dynamics of surfactants, as well as the resulting phase configuration. Such characteristics also impact the macroscopic behavior of the emulsion: under the influence of gravity, water-in-oil emulsions separate more rapidly than oil-in-water systems, highlighting a fundamental difference in the stability and topology of these two system types. Recent studies^6–11^ have highlighted significant differences in droplet size, effective viscosity, and stability between oil-in-water and water-in-oil emulsions, further emphasizing the crucial role that surfactants play in various industrial processes, including emulsion stabilization^12,13^ and froth flotation.^14,15^ For process optimization and improvement, a more detailed understanding of surfactant concentration in both phases and at the interface is highly desirable. However, directly measuring this concentration is challenging, as surfactants are effectively invisible and cannot be directly detected at the interface. This limitation, which adds to the already complex task of experimentally measuring the three-dimensional dynamics of topology-changing, drop-laden flows,^200–202^ makes it difficult to interpret experimental results. Surfactant distributions are usually inferred from observable fluid phenomena, such as velocity fields, free surface dynamics, or Laplace pressure measurements, with only a few studies focused on their dynamics in turbulent conditions.^4^ However, ultimately, the distribution of surfactants within the bulk phases and at the interface remains unclear.

Interface-resolved simulations of multiphase turbulence provide a unique opportunity to obtain detailed space- and time-resolved information on the dispersed phase topology and surfactant concentration. The numerical modeling of surfactants has gained increasing attention due to the development of various methods, which can be coupled with front-tracking,^16,17^ volume-of-fluid,^18,19^ level-set,^20,21^ and phase-field approaches.^22–24^ Generally speaking, surfactants can exhibit two different behaviors: (i) insoluble surfactants, chemical compounds that are present only at the interface while they cannot be adsorbed/desorbed in the bulk of the two phases; (ii) soluble surfactants, chemical compounds that can be adsorbed/desorbed at the interface and in the bulk of one or of both phases.^25^ This distinction, which is required for the numerical modeling of surfactants, determines how surfactant concentration is described in simulations. For insoluble surfactants–which are present only at the interface–a single governing equation is solved either at the interface, in a narrow band around it, or throughout the domain.^26–28^ For soluble surfactants, which are present both at the interface and in the bulk of the phases, three main modeling approaches are commonly used: single-equation models,^22,23^ two-equation models,^29^ and three-equation models.^30^ Single-equation models use a single governing equation to describe the surfactant concentration throughout the system, treating the interface implicitly. Two-equation models solve separately for the surfactant concentration at the interface and for a single bulk concentration field that is continuous across both phases. In this case, source and sink terms are included to model the exchange of surfactant between the bulk and the interface. Finally, three-equation models employ three separate variables to represent the surfactant concentration in the system: one equation governs the interfacial concentration, while two distinct equations describe the bulk concentration in each phase.

In this work, we numerically investigate an oil-in-water emulsion (5% oil −95% water) considering surfactants characterized by different hydrophilic–lipophilic balance (HLB). Three different types of surfactants are considered: (i) lipophilic surfactants (low HLB and oil-soluble); (ii) neutral surfactants (intermediate HLB and soluble in both phases); (iii) hydrophilic surfactants (high HLB and water-soluble). To mimic a realistic emulsion, two fluids with the same density (ρ_w_ = ρ_o_ = ρ) and viscosity (η_w_ = η_o_ = η) are considered. We perform direct numerical simulation of the Navier–Stokes and continuity equations, which are used to describe the flow field in the two phases by means of one-fluid approach. Surfactant effect on interfacial tension is accounted for via an equation of state. A two-order-parameter phase-field method is used to describe both interface topology and surfactant concentration. Specifically, two Cahn–Hilliard-like equations–obtained from a Ginzburg–Landau free energy functional–are used to describe the time evolution of the two order parameters. The first order parameter represents the interface position while the second the surfactant concentration. A skewed term is introduced in the free energy functional to describe the different solubility of surfactants in the two phases.^31^

## Materials and Methods

We consider the flow of two immiscible phases in a rectangular flat channel. To describe the dynamics of the system, we couple direct numerical simulation of the Navier–Stokes equations, used to describe the flow field, with a two-order-parameter phase-field method (PFM), used to describe the oil/water interface and the surfactant concentration.^22,23,32^

### Hydrodynamics

The flow field is obtained by solving a single set of Navier–Stokes (NS) and continuity equations in the entire domain.^32,33^ The effect of interfacial tension is accounted for by introducing a source term in the NS equations. We consider the incompressible flow of two Newtonian fluids (oil and water) with equal density (ρ_w_ = ρ_o_ = ρ) and viscosity (η_o_ = η_w_ = η); the continuity and NS equations can be written in dimensionless form as follows

1 ∇·u=0

2 $$\frac{\partial u}{\partial t}+\nabla \cdot \left(u⊗u\right)=-\nabla p+\frac{1}{Re_{\tau }}\nabla^{2}u+\frac{3}{\sqrt{8}}\frac{Ch}{We}\nabla \cdot \left[f_{\sigma }\left(\psi \right)T_{c}\right]$$

where we recall that **u** = (*u*, *v*, *w*) is the velocity vector and *p* is the pressure field. The shear Reynolds number, Re_τ_ = ρ*u*_τ_ *h*/η, represents the ratio between the inertial and viscous forces while the Weber Number, We = ρ*u*_τ_^2^ *h*/σ_0_, identifies the ratio between the inertial and interfacial tension forces. The Weber number is here defined using the interfacial tension of a surfactant-free interface as a reference. The interfacial tension term–last term at the right-hand side–is defined by the Korteweg tensor, *T*_c_, and the equation of state for interfacial tension, *f*_σ_.^34,35^ The Korteweg tensor accounts for the interfacial forces via a continuum surface stress approach^36^ and it is defined as follows

3 $$T_{c}={\left|\nabla \phi \right|}^{2}I-\nabla \phi ⊗\nabla \phi$$

where ***I*** is the identity matrix. The equation of state (EOS) for the interfacial tension describes the action of surfactant on interfacial tension. We adopt here a modified Langmuir equation of state, *f*_σ_(ψ)^34,35^

4 $$f_{\sigma }\left(\psi \right)=1+\beta_{s}\log\left(1-\psi \right)$$

where β_s_ is the elasticity number that defines the strength of the surfactant effect.

### Phase-Field Method for Hydrophilic–Lipophilic Surfactants

To describe the interface shape, its topological changes and the surfactant concentration in the system, a two-order-parameter PFM is employed.^22,35,37^ The method is here extended to surfactants characterized by different HLB and thus by a different solubility in the two phases. In the framework of the PFM, the interface between the two phases is represented by the first order parameter, ϕ. In the bulk of each phase, the phase field variable has a uniform value (ϕ = – 1 for the continuous phase and ϕ = +1 for the dispersed phase), while across the interface separating them, it transitions continuously from one value to the other. The surfactant concentration is represented by the second order parameter, ψ. The surfactant concentration has a low value in the bulk of the two phases while it reaches its maximum value at the interface where surfactant molecules preferentially accumulate. The starting point for determining the governing equations for the two order parameters is a Ginzburg–Landau free-energy functional, which expression reads as follows

5 $$F\left[\phi ,\nabla \phi ,\psi \right]=\int_{\Omega }\left(f_{0}+f_{m}+f_{\psi }+f_{1}+f_{b}+f_{a}\right)d\Omega$$

where Ω is the domain. The first term, $f_{0}={\left(\phi^{2}-1\right)}^{2}/4$ , describes the tendency of the system to separate into two phases. The second term, *f*_*m*_ = (Ch^2^/2)|**∇**ϕ|^2^, accounts for the energy of the interface, where the Cahn number, Ch, determines the interface thickness. The third term, *f*_ψ_ = *Pi*[ψ ln ψ + (1−ψ) ln (1−ψ)], describes the tendency of surfactant molecules to distribute uniformly, where *Pi* determines the surfactant diffusivity. Surfactant adsorption at the interface is described by the terms $f_{1}=-\left(1/2\right)\psi {\left(1-\phi^{2}\right)}^{2}$ and *f*_*b*_ = (1/2*E*_*x*_)­ψϕ^2^, where *E*_*x*_ determines the surfactant solubility. To extend the model used by Soligo et al.^38^ to account for the hydrophilic/lipophilic character of the surfactant, we introduce a skewed term: *f*_*a*_ = −(*A*_*x*_/2)­ψϕ^3^. The parameter *A*_*x*_ identifies the difference in free energy of dissolving the surfactant in the oil or water phase depending on its HLB. The transport of the two order parameters is governed by two Cahn–Hilliard-like equations, which in dimensionless form read as follow

6 $$\frac{\partial \phi }{\partial t}+u\cdot \nabla \phi =\frac{1}{Pe_{\phi }}\nabla^{2}\mu_{\phi }+f_{p}$$

7 $$\frac{\partial \psi }{\partial t}+u\cdot \nabla \psi =\frac{1}{Pe_{\psi }}\nabla \cdot \left[\psi \left(1-\psi \right)\nabla \mu_{\psi }\right]$$

The two Péclet numbers express the ratio between diffusive and convective time scales. In these equations, the diffusive part is driven by the gradient of the chemical potential of the respective order parameter. The expressions of the chemical potentials are obtained by taking the functional derivatives of the free energy functional

8 $$\mu_{\phi }=\frac{\delta F}{\delta \phi }=\phi^{3}-\phi -{Ch}^{2}\nabla^{2}\phi +C\left(\phi ,\psi \right)$$

9 $$\mu_{\psi }=\frac{\delta F}{\delta \psi }=P_{i}\ln\left(\frac{\psi }{1-\psi }\right)-\frac{1}{2}{\left(1-\phi^{2}\right)}^{2}+\frac{1}{2E_{x}}\phi^{2}-\frac{A_{x}}{2}\phi^{3}$$

The coupling term *C*(ϕ, ψ) is here neglected following the approach employed by Yun et al.,^34^ which assumes that the phase field interfacial profile is independent of the surfactant concentration. The penalty flux *f*_p_, introduced in the profile-corrected formulation,^39^ is used to circumvent some drawbacks present in the original formulation of the phase-field method and is defined as follows

10 $$f_{p}=\frac{\lambda }{Pe_{\phi }}\left[\nabla^{2}\phi -\frac{1}{\sqrt{2}Ch}\nabla \cdot \left(\left(1-\phi^{2}\right)\frac{\nabla \phi }{\left|\nabla \phi \right|}\right)\right]$$

From eqs 8 and 9, the equilibrium profiles for the phase-field and surfactant concentration can be derived analytically. At equilibrium, the respective chemical potentials are uniform throughout the domain, implying that **∇**μ_ϕ_ = 0 and **∇**μ_ψ_ = 0. Considering a planar interface, the resulting equilibrium profile for the phase field is

11 $$\phi_{eq}=\tan h\left(\frac{s}{\sqrt{2}Ch}\right)$$

where *s* is the coordinate normal to the interface while for the surfactant concentration

12 $$\psi_{eq}=\frac{\psi_{b}^{*}}{\psi_{b}^{*}+\psi_{k}\left(\phi_{eq}\right)\left(1-\psi_{b}^{*}\right)}$$

where ψ_b_^*^ is the bulk concentration in the phase with larger solubility (reference bulk concentration). The auxiliary function, ψ_*k*_(ϕ_eq_), is defined as

13 $$\psi_{k}\left(\phi_{eq}\right)=\exp\left\{-\frac{1}{2P_{i}}\left[{\left(1-\phi_{eq}^{2}\right)}^{2}+\frac{1}{E_{x}}\left(1-\phi_{eq}^{2}\right)-\left(\left|A_{x}\right|-A_{x}\phi_{eq}^{3}\right)\right]\right\}$$

The surfactant concentration is uniform in the bulk of the two phases and peaks at the interface, where surfactant molecules accumulate. It is worth noticing that for all cases the concentration peaks at ϕ = 0 due to the inclusion of a term proportional to ϕ^3^ in the energy functional; the use of a term proportional to ϕ would not guarantee this property among the different cases considered. From eq 12, we notice that the surfactant concentration in the two bulk is different, as expected. The ratio between the two bulk concentrations is

14 $$K=\frac{{\psi_{b}}^{+}}{{\psi_{b}}^{-}}=\exp\left(\frac{A_{x}}{Pi}\right)$$

which defines the partition ratio *K*.^40^ Here, ψ_*b*_^+^ is the bulk concentration in the phase characterized by ϕ = +1 (oil droplets) and ψ_*b*_^–^ is the bulk concentration in the phase characterized by ϕ = – 1 (water carrier).

### Numerical Method

The governing equations are solved using a pseudospectral method.^41,42^ All variables are defined in an Eulerian framework and are discretized on a grid with uniform spacing in the *x*- and *y*-directions, while Chebyshev–Gauss–Lobatto points are used in the *z*-direction. Spatial discretization is operated using Fourier series in the streamwise (*x*) and spanwise (*y*) directions, and Chebyshev polynomials in the wall-normal (*z*) direction. Time integration follows an implicit-explicit approach: linear terms are handled with an implicit scheme, and nonlinear terms with an explicit one. The Navier–Stokes equations and continuity equations are first rewritten in the so-called velocity-vorticity formulation.^41,42^ The first Cahn–Hilliard equation (phase-field variable) is split into two second order equations.^43^ Instead, the transport equation for the surfactant concentration is directly solved in its original form. The numerical scheme described above has been implemented in our in-house parallel GPU-ready open-source code named FLOW36.^44^ A closed channel configuration is simulated. Periodicity is implicitly applied in the streamwise (*x*) and spanwise (*y*) directions through the discretization of variables using Fourier series. At the channel walls, no-slip conditions are enforced for the velocity field while for the phase field variable and surfactant concentration fields, and their respective chemical potentials, no-flux boundary conditions are applied.

### Simulation Setup

The computational domain is a closed channel with dimensions *L*_*x*_ × *L*_*y*_ × *L*_*z*_ = 4π*h* × 2π*h* × 2*h* corresponding to *L*_*x*_^+^×*L*_*y*_^+^×*L*_*z*_^+^ = 3770 × 1885 × 600 wall units. A dual grid approach is used to optimize the computational efficiency by using different resolutions for different variables.^44,45^ For the velocity and phase field, a grid with *N*_*x*_ × *N*_*y*_ × *N*_*z*_ = 512 × 256 × 513 points is used to describe the turbulent flow and the interface dynamic. For the surfactant concentration, a finer grid with *N*_*x*_ × *N*_*y*_ × *N*_*z*_ = 2048 × 1024 × 513 points is used; this ensures an accurate resolution of the steep gradients in the surfactant concentration field.

The simulations are conducted at a shear Reynolds number equal to Re_τ_ = 300, computed using the friction velocity $u_{\tau }=\sqrt{\tau_{w}/\rho }$ as a reference. This corresponds to a bulk Reynolds number–computed using the mean channel velocity–of about Re_*b*_ = 5000 and thus to a fully turbulent regime. Two values of the interfacial tension have been considered, defined via the Weber number (We = 1.5 and We = 3.0). We consider two phases with the same density and viscosity. This assumption is made to reduce the complexity of the parameter space and to isolate the effects of surfactant selective solubility. Despite this simplification, the model remains relevant to a broad class of practical oil–water emulsions. A previous study by Mangani et al.^46^ demonstrated that viscosity and density ratios in the range of 0.1 to 10 do not significantly influence droplet topology. As typical oil–water systems exhibit density and viscosity contrasts within this range, we believe that this assumption does not compromise the generality of the results obtained. For the phase field, a Cahn number equal to Ch = 0.025 is used. The phase field Péclet number is set to Pe_ϕ_ = 1/Ch = 40, as recommended by Magaletti et al.,^47^ to achieve the sharp interface limit. For the surfactant concentration, a Péclet number equal to Pe_ψ_ = 100 is adopted.

For each Weber number, we consider five surfactants, which differ for their HLB. We span from a surfactant characterized by HLB = 3.3 (lipophilic; oil-soluble) up to HLB = 16.6 (hydrophilic; water-soluble). Each HLB value can be linked to a specific partition ratio via the following empirical relation^48^

15 log(K)=a−bHLB

where *a* and *b* are constants that are obtained by fitting experimental data (here we employ *a* = 1.5 and *b* = 0.15). The values for *a* and *b* have been chosen to span a broad range of potential scenarios. For example, Span surfactants are usually characterized by HLB in the range from 4 to 8 and have a corresponding partition ratio that ranges from 5 to 100. Differently, Tween surfactants are usually characterized by HLB in the range from 14 to 17 and have a corresponding partition ratio that ranges from 40 to 80. When targeting a specific surfactant chemistry, ad-hoc values can be set for *a* and *b*. Once computed the partition ratio *K* for each HLB, using eq 14 we can map the surfactant HLB to the phase-field parameters. In particular, we can compute the corresponding value of the asymmetric solubility parameter *A*_*x*_. Analyzing Figure 1, we can see that surfactants with low HLB are identified by positive *A*_*x*_ (left) while surfactants with high HLB are identified by negative *A*_*x*_ (right).

**Figure 1:**
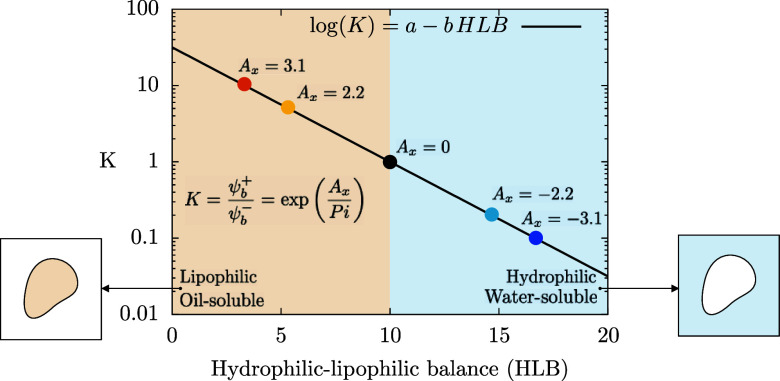
Partition ratio *K* (ratio between the surfactant concentration in the oil over the water phase) as a function of the HLB of surfactants. For a given value of the HLB, the partition ratio can be computed using eq 15. Once the partition ratio *K* is known, the HLB of the surfactant can be mapped to the phase-field parameter that controls phase-selective solubility *A*_*x*_.

We investigate three scenarios: (i) oil-soluble (OS) surfactant cases, where surfactant is soluble in the oil droplets; (ii) equally soluble (ES) surfactant cases, where solubility is equal in both phases; (iii) water-soluble (WS) surfactant cases, where surfactant is soluble in the water carrier phase. For the OS cases (low HLB), surfactant is soluble in the oil droplets where its reference bulk concentration is equal to ψ_b_^*^ = ψ_*b*_^+^ = 0.01. We consider HLB = 3.3 and HLB = 5.3 corresponding to *A*_*x*_ = 3.1 (*K* = 10) and *A*_*x*_ = 2.2 (*K* = 5). The resulting concentration in the water carrier phase can be computed as ψ_*b*_^–^ = ψ_*b*_^+^/*K*. For the ES cases (intermediate HLB), we obtain *A*_*x*_ = 0 and the reference bulk surfactant concentration is ψ_b_^*^ = ψ_*b*_^+^ = ψ_*b*_^–^ = 0.01 in both phases (*K* = 1). Finally, for the WS cases (high HLB), the reference bulk concentration in the water carrier phase is set equal to ψ_b_^*^ = ψ_*b*_^–^ = 0.01. We consider HLB = 14.6 and HLB = 16.6 corresponding to *A*_*x*_ = – 2.2 (*K* = 0.2) and *A*_*x*_ = – 3.1 (*K* = 0.1). The resulting concentration in the oil droplets is ψ_*b*_^+^ = *K*ψ_*b*_^–^. For all cases, *Pi* = 1.35 is kept constant, while *E*_*x*_ is adjusted to maintain the same concentration at the interface at equilibrium. Please refer to Table 1 for an overview of the simulation parameters.

**Table 1:**
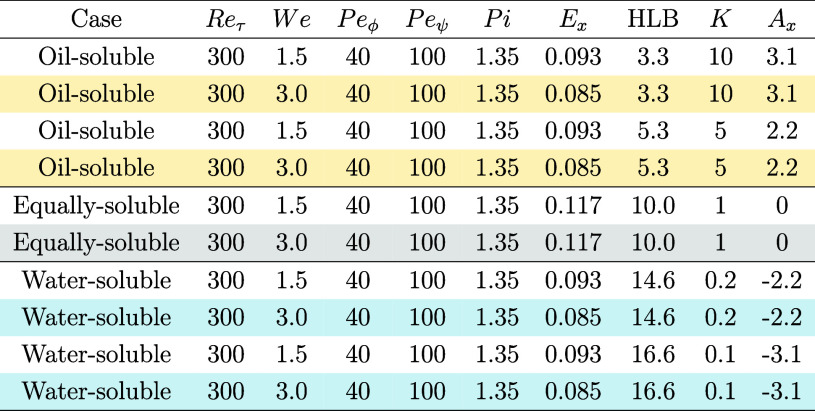
Overview of the Simulation Parameters^a^

a Three series of simulations have been performed: oil-soluble surfactants (HLB <10), equally-soluble surfactant (HLB = 10), and water-soluble surfactants (HLB >10).

The simulations are initialized with a fully developed turbulent channel flow obtained from a single-phase simulation at Re_τ_ = 300. The phase field is initialized with a regular array of 256 spherical droplets of diameter *d* = 0.4*h*, resulting in a dispersed phase volume fraction of approximately Φ ≃ 5.4%. Both the phase field and surfactant concentration are initialized with their equilibrium profiles, eqs 11 and 12, respectively.

## Results and Discussion

In the following, we discuss the results obtained as a function of the asymmetric solubility simulation parameter *A*_*x*_, from smaller to larger values. We recall that *A*_*x*_ is inversely proportional to the HLB and cases with negative *A*_*x*_ correspond to water-soluble surfactants (high HLB) while positive *A*_*x*_ identify surfactants soluble in the oil droplets (low HLB).

### Qualitative Analysis

The three scenarios studied are graphically summarized in Figure 2. The top row shows a contour map of the surfactant concentration obtained from the three configurations: water-soluble (WS, *A*_*x*_ < 0, left column), equally soluble (ES, *A*_*x*_ = 0, central column), oil-soluble (OS, *A*_*x*_ > 0, right column). The surfactant concentration in the system is rendered with a white/black color bar. For the WS cases (left), low concentration values are obtained inside the dispersed phase while larger values are obtained in the carrier phase (gray) and of course, much larger values are obtained at the interface where surfactant accumulates. By opposite, for the OS cases (right), low concentrations are obtained in the carrier while larger values are obtained inside the droplet and much larger values are obtained at the interface (black). The equilibrium profiles for *A*_*x*_ = – 3.1 (left), *A*_*x*_ = 0 (center) and *A*_*x*_ = 3.1 (right) are also reported in the bottom row.

**Figure 2:**
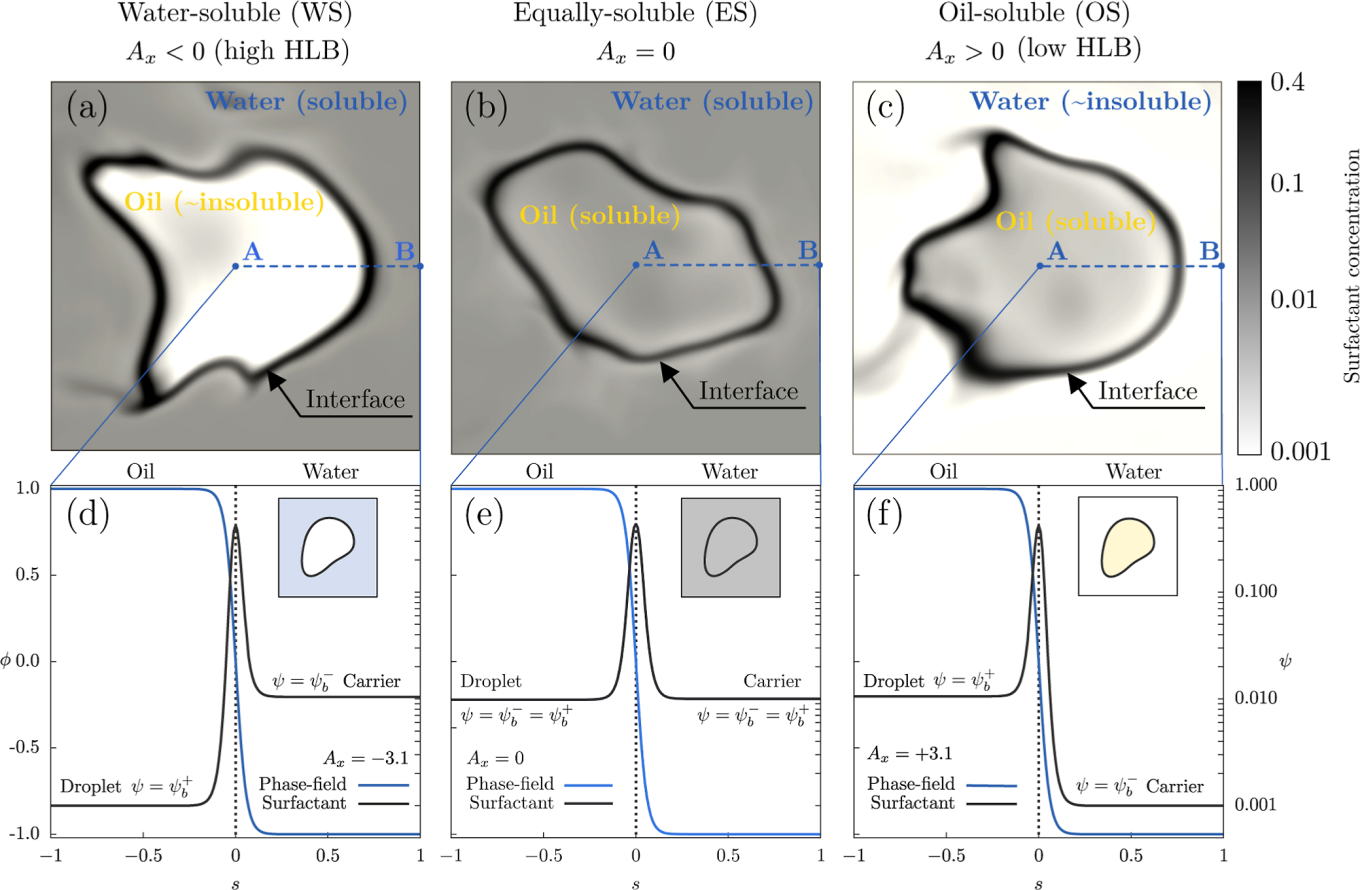
Representation of the three scenarios considered: water-soluble (left, panels *a* and *d*), equally soluble (center, panels *b* and *e*), oil-soluble (right, panels *c* and *f*). The top row shows a contour map of the surfactant concentration profile in a plane that crosses one drop (white-low; black-high) while the bottom row shows the surfactant concentration along the dashed line. For the WS cases, the concentration inside the oil droplets is lower (white) with respect to the water carrier (gray). Differently, for the OS cases, the concentration in the water carrier is lower (white) than the one inside the droplets (gray).

We compare now from a qualitative point of view the simulation results. All the simulations exhibit an initial transient stage. During this stage, the dispersed phase evolves from the initial condition toward a steady state and at the same time the surfactant distributes in the system and reaches a new equilibrium configuration. The evolution of the interfacial behavior (droplets) and surfactant distribution are characterized by complex dynamics and many phenomena influence the final steady-state configuration.^49^ The coupling among these phenomena is graphically summarized in Figure 3. Starting from the left, changes in the interfacial area lead to modifications in the interfacial surfactant concentration and thus in the interfacial forces. These changes have direct consequences on breakage and coalescence events: the first are favored by high surfactant concentrations (low interfacial tension values) while uneven surfactant concentrations, which in turn lead to the generation of Marangoni stresses, can hinder coalescence. Topological changes (breakage and coalescence) in turn feedback on the amount of interfacial area: breakage events increase the interfacial area while coalescence events lead to a reduction of the interfacial area.

**Figure 3:**
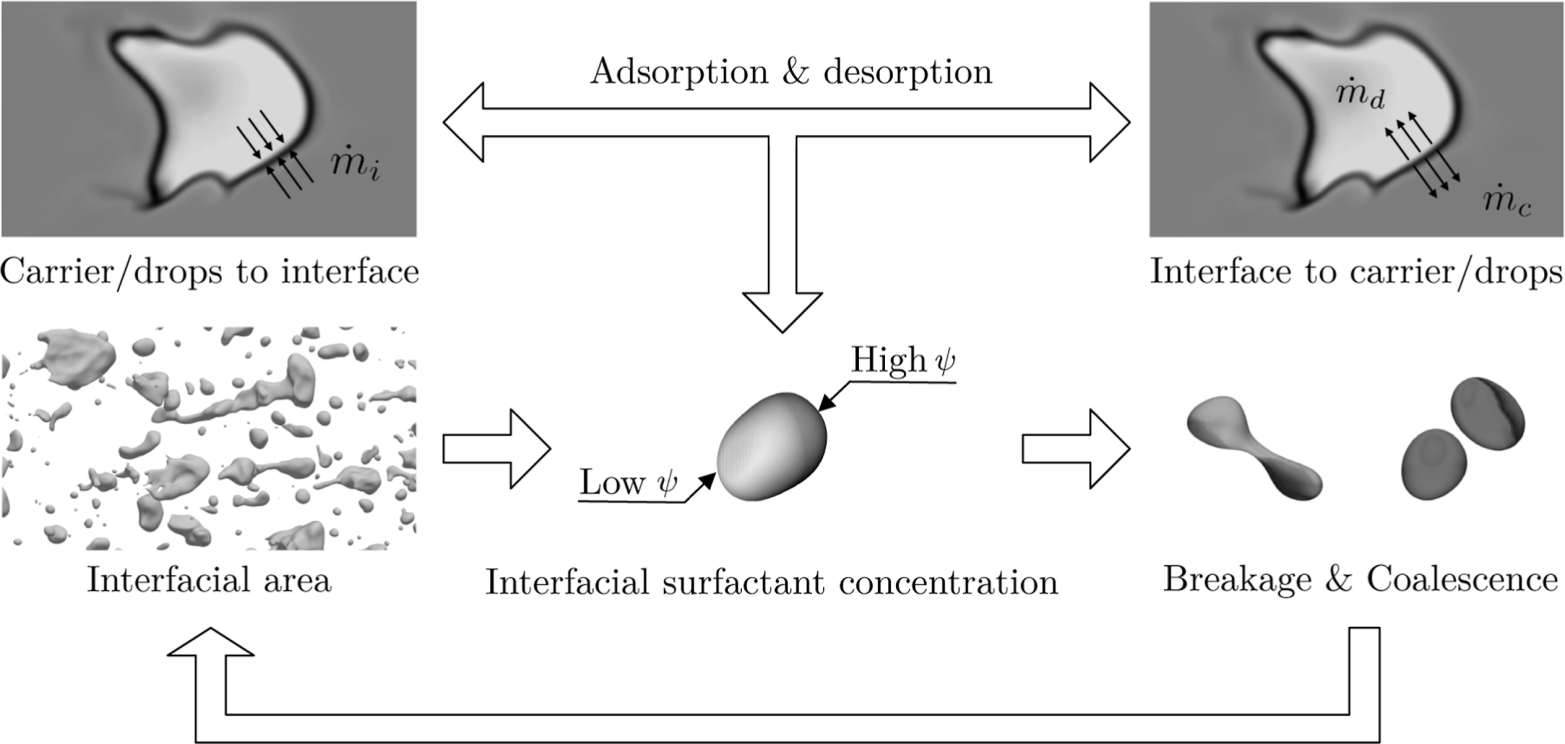
Sketch that illustrates the main factors controlling the distribution of the surfactant in the multiphase system. Starting from the bottom left, the amount of interfacial area is the leading factor in determining the average surfactant concentration at the interface. Changes of the interfacial concentration, which change the average value of the surface tension, can promote breakage and coalescence events and thus modifications of the amount of interfacial area (increase and decrease, respectively). In addition to this feedback mechanism, modifications of the interfacial surfactant concentration and thus nonequilibrium conditions also lead to adsorption and desorption fluxes from the bulk of the two phases.

Further complexity arises from the adsorption and desorption of surfactant between the interface and the bulk of the two phases, as illustrated in the upper part of Figure 3. When the surfactant concentration at the interface exceeds its equilibrium value, thermodynamic drives desorption into the bulk to reduce the system free energy. Conversely, when the concentration falls below equilibrium, surfactant molecules adsorb from the bulk onto the interface. These exchanges become even more intricate when the two phases exhibit different solubilities, leading to an asymmetric adsorption/desorption process, where surfactant molecules selectively adsorb or desorb into one phase over the other.

To appreciate these differences in the surfactant distribution, we can consider Figure 4, which shows the surfactant concentration in a *x* ^+^ – *y* ^+^ plane located at the channel center (*t* ^+^ = 5010). All cases shown refer to *We* = 3.0. Each row refers to a different selective solubility scenario: the top row refers to the carrier-soluble case (WS, *A*_*x*_ = – 3.1), panel *b* to the equally soluble case (ES, *A*_*x*_ = 0) while panel *c* refers to the droplet-soluble case (OS, *A*_*x*_ = +3.1). Starting from panel *a* (WS), we can identify the interface shape, which is characterized by a black color (high concentration) and it is where surfactant molecules preferentially accumulate. Moving away from the interface, we observe that the bulk of the two phases is characterized by different shades of gray. Clearly, the surfactant concentration is lower with respect to the interface. Nevertheless, we can appreciate some differences and surfactant concentration is larger in the bulk of the carrier (gray, soluble phase) while the region inside the droplets is characterized by a lighter color (almost white, insoluble phase). This is expected, as we are considering the WS case, where surfactant is soluble in the carrier. It is worth noticing that surfactant concentration in the bulk of the phases is rather uniform, though some small variations can be observed in both the carrier and dispersed phases. Moving to panel *b* (ES), the interfacial region is again characterized by a dark, almost black color. Comparing the bulk of the two phases, the concentrations are now very similar and have similar colors (gray). This is coherent with the fact that carrier phase and droplets are characterized by an equal solubility. However, some minor differences can be still appreciated, and surfactant concentration inside the droplets seems slightly smaller than that in the carrier phase. Finally, we consider panel *c* (OS), where the situation is the opposite compared to that observed in panel *a*. In particular, it is clear that the concentration inside the droplets (gray, soluble phase) is larger than that observed in the carrier phase (light gray). The surfactant concentration in the bulk of the two phases is rather uniform, though some variations can be appreciated.

**Figure 4:**
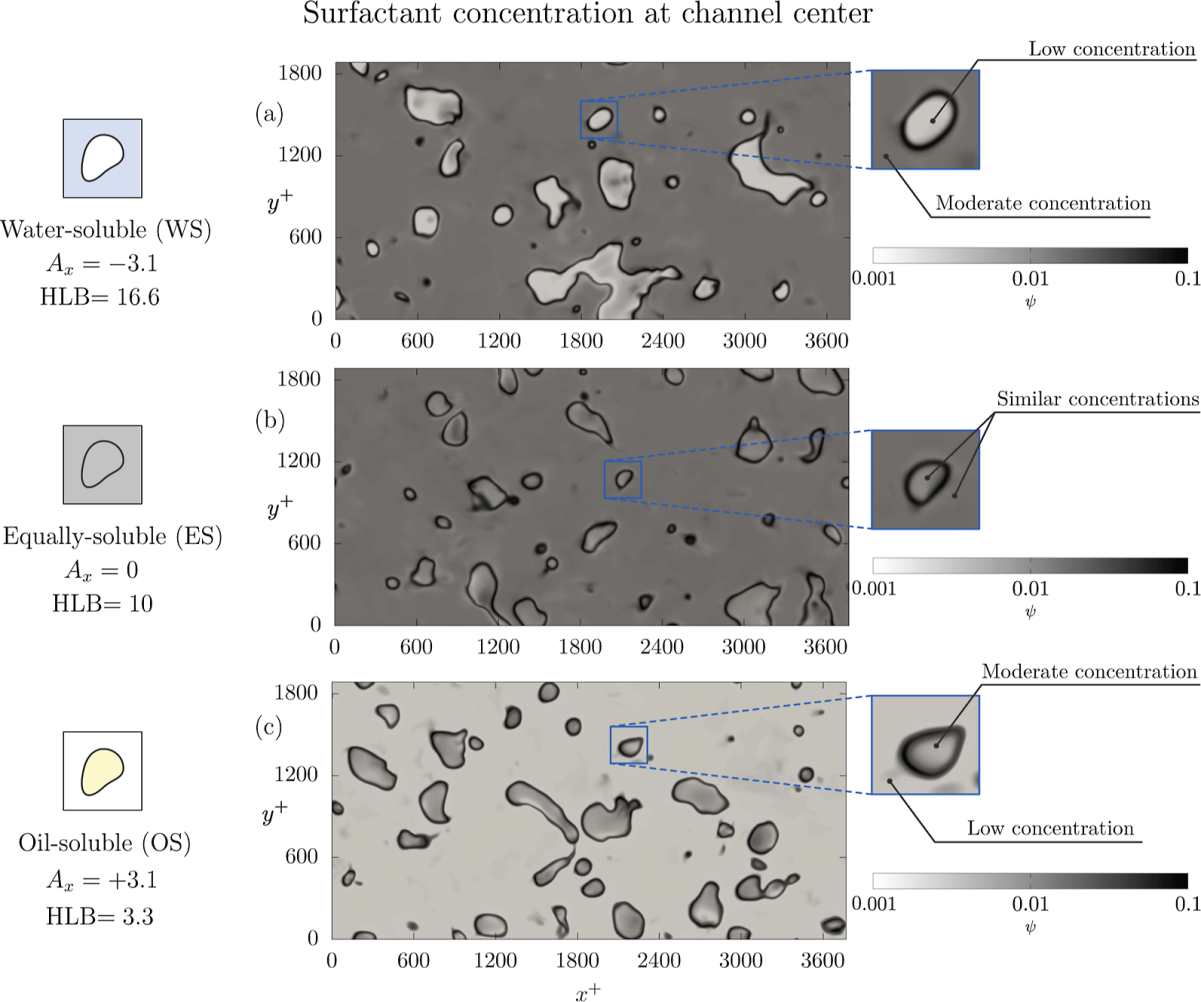
Contour map of the surfactant concentration (white-low; black-high) in a *x* ^+^ – *y* ^+^ located at the channel center at *t* ^+^ = 5010 for We = 3.0. Each panel corresponds to a different case: panel *a* to the water-soluble case (*A*_*x*_ = – 3.1); panel *b* to the equally soluble case (*A*_*x*_ = 0) and panel *c* to the oil-soluble case (*A*_*x*_ = +3.1). The close-up views reported on the right side allow to appreciate the different concentrations obtained in the oil and water phases for the different *A*_*x*_ considered.

Let us consider the surfactant concentration at the interface. Figure 5 shows the interface of the droplets (iso-contour ϕ = 0) colored by the surfactant concentration (white-low; black-high). The colormap is now centered on larger values so that variations on the droplet surface can be better appreciated. The three panels refer to the same time instants shown in Figure 4 and the same Weber number (*We* = 3.0). Each row refers to a different selective solubility scenario: panel *a* refers to the carrier-soluble case (WS, *A*_*x*_ = – 3.1), panel *b* to the equally soluble case (ES, *A*_*x*_ = 0) and panel *c* to the droplet-soluble case (OS, *A*_*x*_ = 3.1). From panel *a*, we can observe many droplets of very different sizes, from small spherical droplets to larger drops, characterized by very complex shapes. Also, we can appreciate that the surfactant concentration at the interface is uneven, and black regions, characterized by high values of the surfactant concentration (low interfacial tension), are alternated to gray/white regions where surfactant concentration is lower (high interfacial tension), as shown in the close-up view of panel *a*. The uneven surfactant concentration obtained at the interface of the droplets leads to an uneven value of the interfacial tension on the surface of the droplets. This, in turn, generates Marangoni stresses, which are tangential to the droplet interface and proportional to the interfacial tension gradients. Moving to panel *b*, the situation is not much different with respect to panel *a*: the topology of the dispersed phase is similar as well as the range of concentrations observed on the surface of the droplets. Finally, moving to panel *c*, while the topology of the dispersed phase remains similar to that observed in the previous panels, the surface of the droplets is characterized by darker shades of colors, with respect to that obtained in panels *a*, *b*. This seems to suggest that the range of surfactant concentrations observed is larger and, in turn, this indicates a larger interfacial tension reduction.

**Figure 5:**
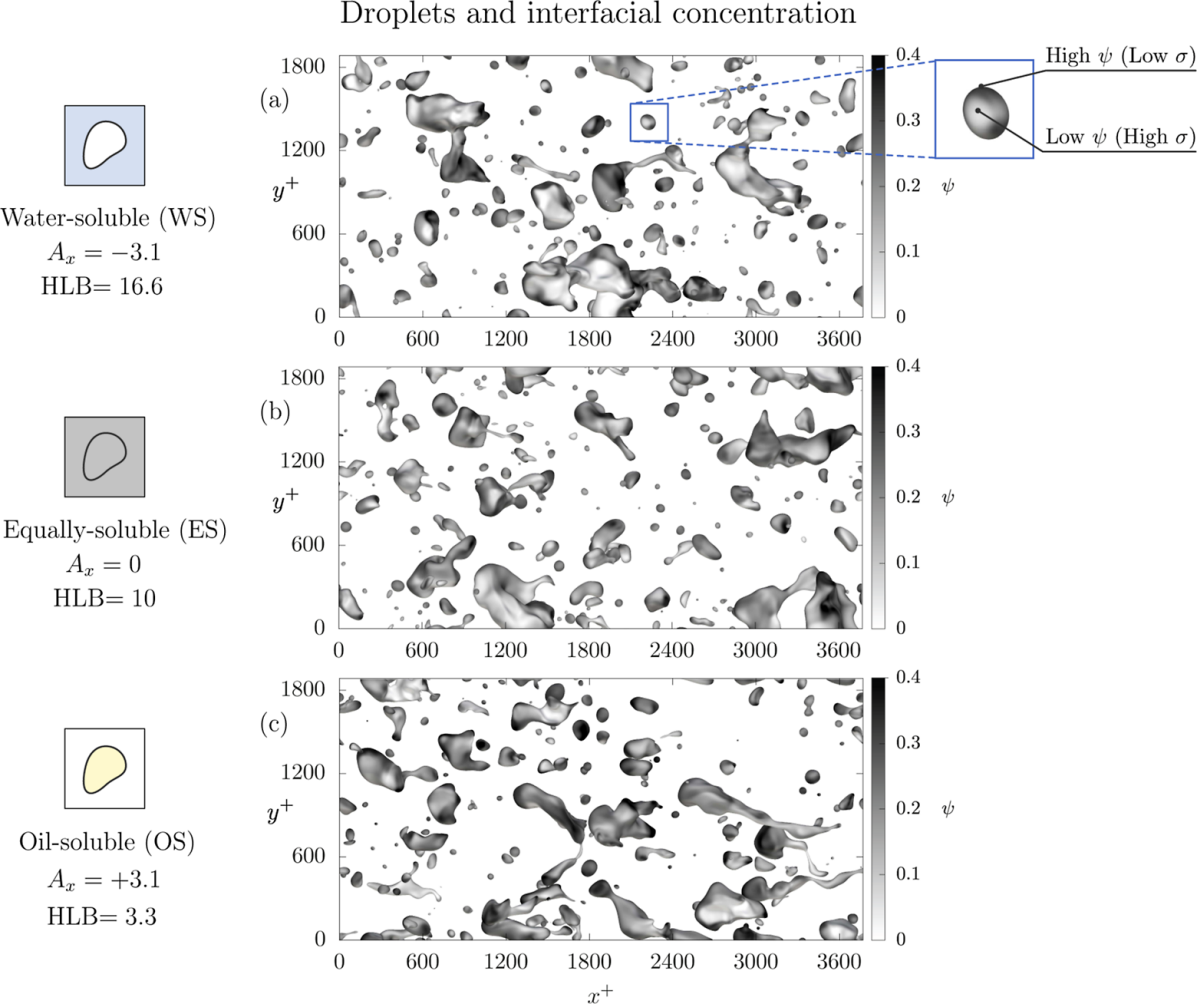
Top view of the steady-state configuration attained at *t* ^+^ = 5010 for the cases at We = 3.0. The interface of the droplets (iso-contour ϕ = 0) is colored by the surfactant concentration at the interface (white-low; black-high). Each row corresponds to a different case: panel *a* refers to the water-soluble cases (*A*_*x*_ = – 3.1), panel *b* refers to the equally soluble cases (*A*_*x*_ = 0) while panel *c* refers to the oil-soluble cases (*A*_*x*_ = 3.1). The uneven surfactant concentration generates a non-uniform interfacial tension on the surface of the droplets, as shown in the close-up view.

### Droplet Size Distribution

To quantify the previous qualitative observations, we analyze the dispersed phase topology. The first observable that we consider is the droplet size distributions (DSD) obtained at steady-state in the different cases. The DSD is an information on key importance in the modeling of multiphase flow.^50^ Indeed, once known the distribution, important parameters like the amount of interfacial area, which has an important role in heat and mass transfer problems, can be evaluated. An important length scale when evaluating the drop size distribution is the Kolmogorov-Hinze diameter.^51^ This diameter represents the maximum stable diameter for a nonbreaking droplet. This diameter can be calculated from the balance between the destabilizing forces that act on the droplet surface (e.g., turbulence-induced stresses) and the stabilizing action of interfacial tension, which tries to minimize the droplet surface and restore the spherical shape thus avoiding droplet breakage. For the present configuration (turbulent channel flow), this diameter can be estimated as follows^35^

16 $${d_{H}}^{+}=0.725{\left(\frac{\sigma_{0}}{\sigma_{av}}\frac{We}{Re_{\tau }}\right)}^{-3/5}{\left|\epsilon_{c}\right|}^{-2/5}$$

The ratio σ_av_/σ_0_ represents the average reduction in interfacial tension caused by the presence of surfactant and is approximately σ_av_/σ_0_ ≃ 0.7 for all cases. The term ϵ_*c*_ denotes the turbulent dissipation evaluated at the channel center, where droplets preferentially accumulate. For a fixed Reynolds number, higher Weber numbers correspond to weaker interfacial tension forces, leading to smaller maximum stable droplet diameters. Specifically, the resulting Kolmogorov-Hinze scales are *d*_*H*_^+^≃165 *w*.*u*. for We = 1.5 and *d*_*H*_^+^≃110 *w*.*u*. for We = 3.0. For the OS cases, the Kolmogorov-Hinze scale is slightly smaller due to the higher surfactant concentration at the interface, as qualitatively illustrated in Figure 5.

Figure 6 shows the droplet size distributions obtained from the different cases. The DSDs have been computed from *t* ^+^ = 3000 up to *t* ^+^ = 6000 (steady-state condition for the interfacial area). Results at We = 1.5 are reported with full circles while those at We = 3.0 with empty circles. The different scenarios considered are reported with different colors: WS cases (*A*_*x*_ < 0) in cyan/blue; ES cases (*A*_*x*_ = 0) in black and OS cases (*A*_*x*_ > 0) in yellow/orange. The analytic scaling laws for the coalescence- and breakage-dominated regimes, *d* ^+–3/2^ and *d* ^+–10/3^, are also reported as a ref 52. Results are shown normalized by the Kolmogorov-Hinze scale of each case. Present results are also compared with archival literature data on DSD obtained in previous works that investigated the breakage of drops/bubbles in turbulent flows. In particular, the following results are reported: breakage of surfactant-laden drop in homogeneous isotropic turbulence;^24,53^ breakage of surfactant-laden drop in turbulent channel flow^35^ breakage of clean drops in homogeneous isotropic turbulence.^54^

**Figure 6:**
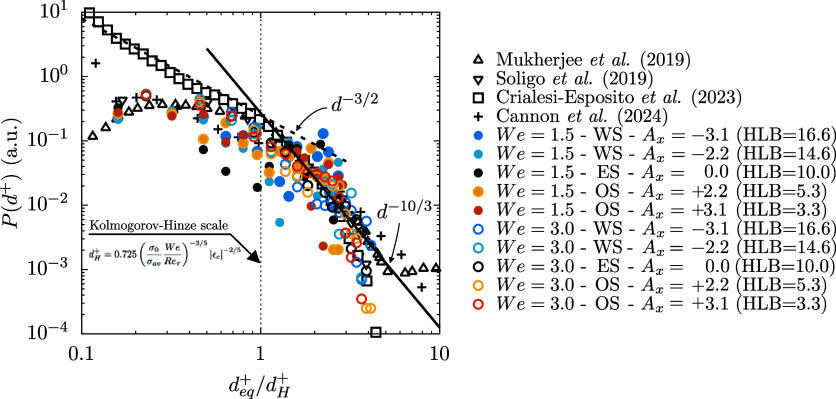
Probability density function of the droplets equivalent diameter *d*_*eq*_^+^ normalized by the Kolmogorov-Hinze scale. Results at *We* = 1.5 are reported with full circles while those at *We* = 3.0 with empty circles. The analytic scaling laws for the coalescence- and breakage-dominated regimes, *d* ^+–3/2^ and *d* ^+–10/3^, are also reported as a reference. A good agreement is obtained in the breakage-dominated regime, i.e., for drops larger than the Kolmogorov-Hinze scale.

Analyzing Figure 6, we can observe the emergence of two regimes depending on the droplet diameter considered. For droplets smaller than the Kolmogorov-Hinze scale, we observe the coalescence-dominated regime (left). In this regime, drops are unlikely to break, as they are smaller than the critical scale; instead, they are more prone to change their size by coalescence with other drops. For droplets larger than the Kolmogorov-Hinze scale, we can identify the breakage-dominated regime, where the main mechanism by which drops change their size is via breakage. We can observe that all data obtained from present simulations roughly follows the scaling law in the breakage regime, with a better agreement obtained for the cases at *We* = 3.0, thanks to the higher number of samples (droplets). For the coalescence regime (droplets smaller than the Kolmogorov-Hinze scale), it is difficult to identify a common trend due to the low number of very small droplets available. Nevertheless, for *We* = 3.0 a fairly satisfactory agreement can be appreciated with the corresponding scaling law although the range of diameter for which it is observed is limited. Overall, no significant changes in the DSD are observed when different types of surfactants are considered.

### Interfacial Area

In turbulent flows, droplets are constantly deformed by turbulence-induced stresses, which stretch them and can eventually cause breakage thus increasing interfacial area extension. On the opposite, coalescence events reduce the amount of interfacial area. To characterize the outcome of this competition, we compute the time evolution of the interfacial area extension.

Figure 7 shows the evolution of the total interface area *A* over time, normalized by the initial value *A*_0_. Panel *a* refers to the WS cases while panel *b* to the OS ones. The WS cases are reported in panel *a* using blue colors, while the OS cases are reported in panel *b* with yellow/orange colors. The ES cases are shown with black curves in each panel, as reference. We observe that for all cases, after an initial transient (gray box, Δ*t* ^+^ ≃ 1500) during which the dispersed phase topology reaches the new steady-state configuration, all curves stabilize around a steady-state value, which is different from the initial one. Considering the effect of the Weber number, we observe that simulations with We = 3.0 stabilize with interface areas that are notably larger than those for We = 1.5. In particular, for We = 1.5, all simulations exhibit a steady-state value of the interfacial area equal to *A*/*A*_0_ ≃ 0.4, while for We = 3.0 the resulting steady-value is equal to *A*/*A*_0_ ≃ 0.7. This is due to the larger drop fragmentation obtained at We = 3.0 (lower interfacial tension) and thus the larger number of smaller droplets obtained. In general, we observe that modifying the surfactant HLB does not significantly affect the time evolution of the interface area. However, it is important to note that for the OS cases, the steady-state values of the interfacial area exhibit significant differences, which vary nonmonotonically with HLB.

**Figure 7:**
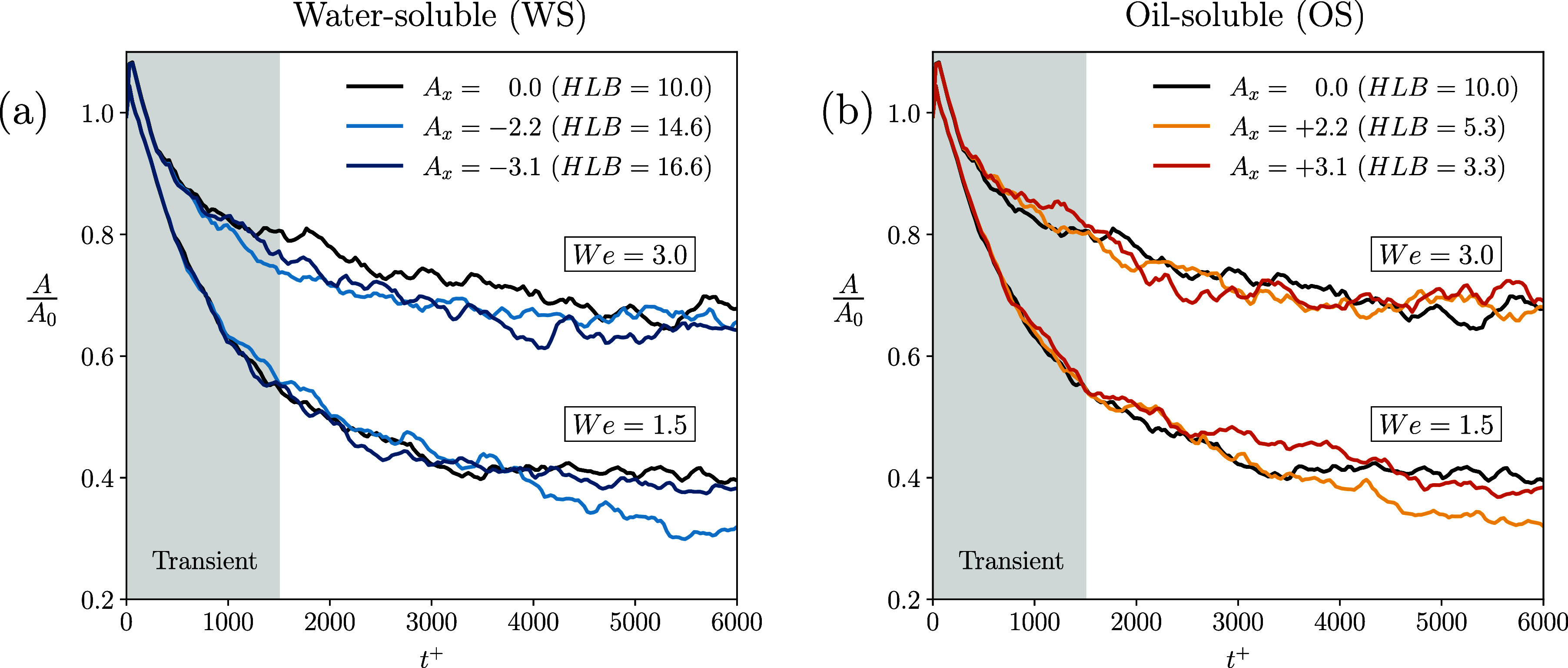
Time evolution of the interface area, *A*, normalized by the initial value, *A*_0_. The panels illustrate the effect of the asymmetric solubility parameter *A*_*x*_. The left panel refers to the water-soluble surfactants while the right panel to the oil-soluble surfactants. Both panels include results for *We* = 1.5 and *We* = 3.0. The equally soluble (ES) surfactant cases are shown with black curves in each panel.

### Surfactant Concentration at the Interface

As shown in Figure 3, surfactant dynamics are closely linked to changes in emulsion morphology and local variations in surface tension, which are influenced by both surfactant concentration and fluxes at the interface and in the bulk. When selective solubility is present, surfactants can preferentially adsorb or desorb from the interface to one phase (WS and OS cases), further complicating the situation by locally altering surface tension. We now focus on how the surfactant HLB impacts interfacial tension and the adsorption/desorption balance, starting with an evaluation of the average surfactant concentration at the interface. Figure 8 shows the mean value of the interfacial surfactant concentration ⟨ψ_*i*_⟩ at steady state. Results are shown normalized by the initial interface concentration ψ_*i*,0_, which is the same across all simulations. Results at *We* = 1.5 are shown using filled symbols while those at *We* = 3.0 with empty symbols. Error bars are used to show the RMS of the surfactant concentration at the interface. Negative values of *A*_*x*_ (left) identify the WS cases while positive values (right) the OS cases.

**Figure 8:**
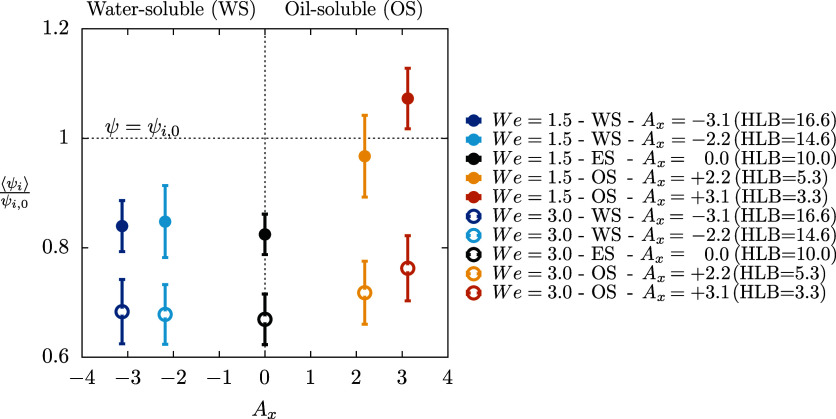
Steady-state value of the surfactant concentration at the interface, ⟨ψ_*i*_⟩, normalized by the initial value ψ_*i*,0_ as a function of the solubility parameter *A*_*x*_. Simulations at *We* = 1.5 are shown using full symbols while those at *We* = 3.0 with empty symbols. Error bars are used to show the spatial RMS of the surfactant concentration. Cases on the left (*A*_*x*_ < 0) represent the water-soluble cases; those on the right (*A*_*x*_ > 0) the oil-soluble cases while *A*_*x*_ = 0 is the equally soluble case.

Comparing the results obtained at the two Weber numbers, we observe that the values attained by the cases at *We* = 3.0 are lower compared to those attained by the simulations at *We* = 1.5. This difference is due to the larger interfacial area at *We* = 3.0, which results from the lower interfacial tension. As a result, the same amount of surfactant (with a small bulk concentration) is spread over a larger surface, leading to a lower average surfactant concentration at *We* = 3.0 (empty symbols) compared to *We* = 1.5. First, consider the hydrophilic/lipophilic cases (*A*_*x*_ ≠ 0), where the surfactant can dissolve effectively in only one phase - either the carrier or the dispersed phase, depending on the sign of *A*_*x*_. The results show that changes in *A*_*x*_ (and thus in the HLB) affect the average surfactant concentration at the interface.

This difference is evident when comparing the reference ES cases (*A*_*x*_ = 0, center) with the WS cases (*A*_*x*_ < 0, left) and OS cases (*A*_*x*_ > 0, right). For the WS cases, only marginal differences are observed, with surfactant concentrations similar to those in the ES cases. In contrast, for the OS cases, the average surfactant concentration increases as *A*_*x*_ increases, as seen in Figure 4 *c*. This trend holds for both Weber numbers and is more pronounced for the smaller Weber number (filled symbols). It is noteworthy that for the larger values of *A*_*x*_ considered, the surfactant concentration at the interface becomes more than 10% higher than the initial concentration, and almost 30% higher than in the ES cases. Despite this increase, no significant changes are observed in the dispersed phase topology. This can be attributed to the fact that the simulation results are based on relatively low Weber numbers, where surface tension dominates. As a result, much larger variations in interfacial tension (compared to those observed here, i.e., σ_*av*_/σ_0_ ≃ 0.7) are needed to induce significant changes in the equivalent Weber number.

### Surfactant Adsorption and Desorption Rates

To understand the mechanisms controlling surfactant concentration at the interface, we analyze the adsorption/desorption dynamics by studying mass transfer rates between the interface and the bulk phases. We define three regions: the carrier phase (ϕ < – 0.95), the interfacial region (|ϕ| < 0.95), and the dispersed phase (ϕ > 0.95), allowing us to measure surfactant transfer rates between the interface, the carrier phase, and the dispersed phase. Since the total surfactant amount in the entire domain Ω is constant, we can now split it into the three subdomains that correspond to the three regions previously defined and we can determine the transfer rates. From the material balance we have

17 $$\int_{\Omega }\psi ,d\Omega =\underset{\Psi_{c}}{\underset{︸}{\int_{\Omega_{c}}\psi ,d\Omega_{c}\;}}+\underset{\Psi_{i}}{\underset{︸}{\int_{\Omega_{i}}\psi ,d\Omega_{i}\;}}+\underset{\Psi_{d}}{\underset{︸}{\int_{\Omega_{d}}\psi ,d\Omega_{d}\;}}=\Psi_{c}+\Psi_{i}+\Psi_{d}=const.$$

We can now evaluate these three contributions at the time step *n* and *n* + 1, and we can write the following conservation equation

18 $$\Psi_{c}^{n}+\Psi_{i}^{n}+\Psi_{d}^{n}=\Psi_{c}^{n+1}+\Psi_{i}^{n+1}+\Psi_{d}^{n+1}$$

By collecting all terms on the left-hand side and dividing by the Δ*t* between two time steps and by the interfacial area (averaged between the time steps *n* and *n* + 1), we obtain

$$\underset{{ṁ}_{c}}{\underset{︸}{\frac{\Psi_{c}^{n+1}-\Psi_{c}^{n}}{\Delta t\left(A^{n}+A^{n+1}\right)/2}}}+\underset{{ṁ}_{i}}{\underset{︸}{\frac{\Psi_{i}^{n+1}-\Psi_{i}^{n}}{\Delta t\left(A^{n}+A^{n+1}\right)/2}}}+\underset{{ṁ}_{d}}{\underset{︸}{\frac{\Psi_{d}^{n+1}-\Psi_{d}^{n}}{\Delta t\left(A^{n}+A^{n+1}\right)/2}}}=0$$ 19

which are the three transfer rates.^55,56^ The resulting balance equation reads as follows

20 $${ṁ}_{c}+{ṁ}_{i}+{ṁ}_{d}=0$$

We can now compute these rates to study how surfactant is transported in the multiphase system.

Figure 9 shows the time evolution of the three transfer rates for all the cases considered. The left panel refers to the WS cases while the right panel displays the OS cases. The top row refers to the simulations at *We* = 1.5 while the bottom row to the simulations at *We* = 3.0. The ES cases results are shown with black curves, as a reference. The squared lines correspond to the carrier phase transfer rate $\left({ṁ}_{c}\right)$ , the circled lines correspond to the dispersed phase transfer rate $\left({ṁ}_{d}\right)$ and the continuous lines identify the interface transfer rate $\left({ṁ}_{i}\right)$ . Positive values of the transfer rates identify a gain of surfactant for the respective region (e.g., carrier, droplets or interface) while negative values of the transfer rates identify a loss of surfactant for the respective region. The time span considered is between *t* ^+^ > 0 and *t* ^+^ < 1500, during which the interfacial area decreases more rapidly, causing surfactant to be redistributed into the two bulk phases.

**Figure 9:**
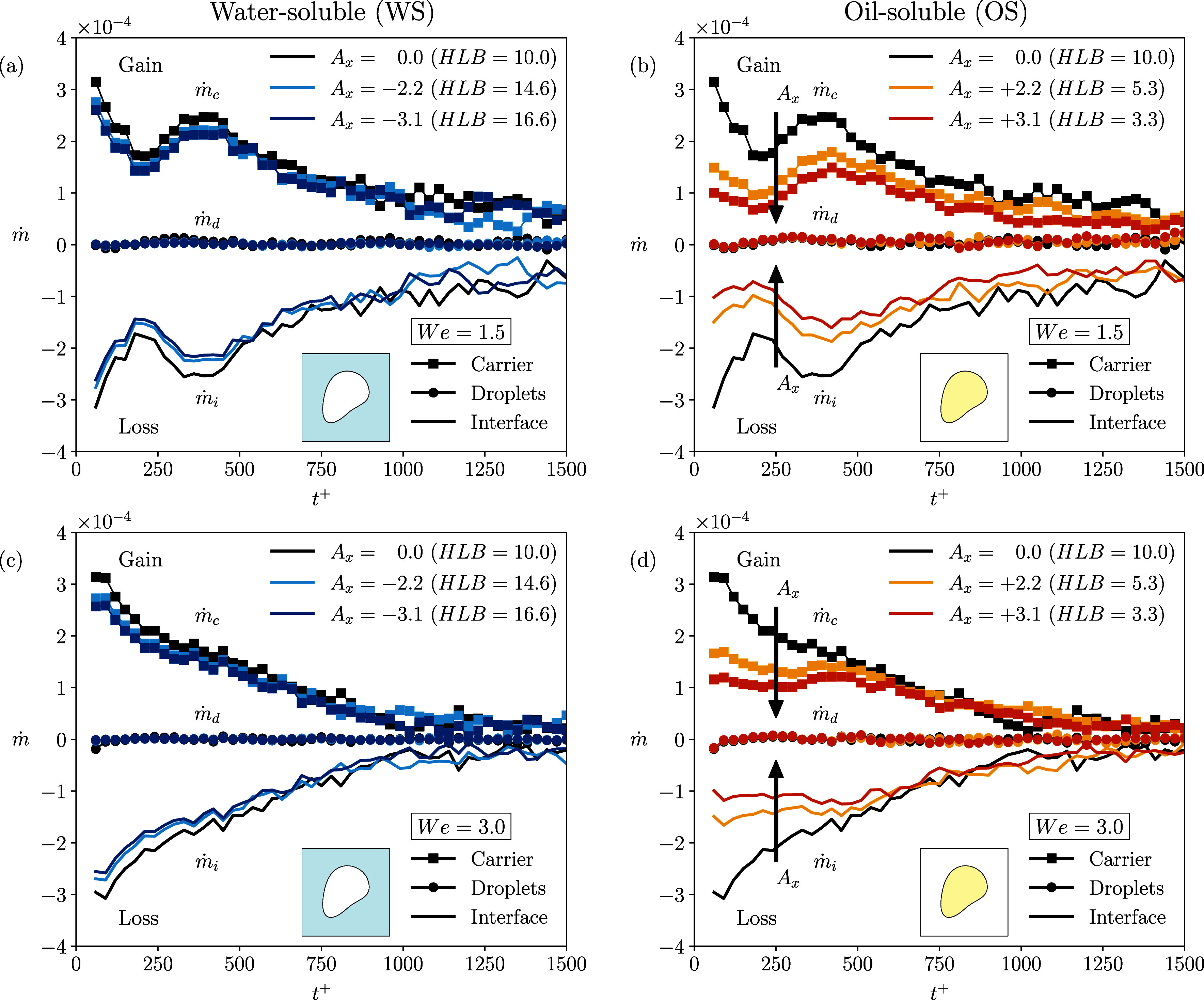
Time evolution of surfactant transfer rates: ${ṁ}_{c}$ (squares), ${ṁ}_{d}$ (circles), and ${ṁ}_{i}$ (line), for the different cases analyzed. The left column illustrates the water-soluble surfactant cases, while the right column refers to the oil-soluble surfactant cases. Equally soluble surfactant cases are shown in black in all panels as a reference. The top row (panels *a* and *b*) corresponds to simulations at *We* = 1.5 while the bottom row (panels *c* and *d*) to *We* = 3.0. Positive values of the transfer rates identify a gain of surfactant for the respective region while negative values identify a loss of surfactant for the respective region.

Initially, the surfactant desorbs from the interface (continuous line) due to the interfacial area reduction until this desorption slows down and eventually stops for later times (*t* ^+^ > 1500). This behavior can be appreciated for both *We* = 1.5 and *We* = 3.0 and across all cases studied. This suggests that the direction of the mass transfer of surfactant is not influenced by the Weber number, nor by the steady-state configuration of the dispersed phase. We can observe that drops only absorb small amounts of surfactant, while the carrier phase absorbs nearly all of the surfactant that desorbs from the interface. Specifically, the interface-to-drops transfer rate (circled lines) remains close to zero, while the interface-to-carrier transfer rate (represented by the squared line) exhibits the same trend of the interface transfer rate (with opposite sign). This indicates a net transfer of surfactant from the interface to the carrier phase. Comparing the WS cases (left) against the OS cases (right), we observe that in the WS cases the carrier phase absorbs almost all surfactant depleted from the interface. Surprisingly, for the OS cases where the opposite is expected (i.e., drops absorbing all surfactant depleted from the interface), the amount of surfactant that the drops absorb is small and a consistent amount of surfactant is found in the carrier phase, tough the amount transferred is smaller compared to the previous WS cases. This observation is in agreement with the increased interfacial concentration attained for the OS cases when *A*_*x*_ > 0 is considered.

This suggests that the carrier phase, rather than the drops, is the phase where surfactant molecules–depleted from the interface by the carrier turbulent flow–accumulate. This observation can be traced back to two main factors. First, the carrier phase forms a connected domain, whereas the drops do not. Second, the carrier phase is in a fully turbulent state whereas the turbulence intensity in the drops is smaller as the structures develop in a confined space and the interface further modulates the energy exchanges with the carrier.^46^ As a result, the carrier has a greater capacity of depleting surfactant from the interface.

To better understand the modulation of the interface-to-carrier transfer rate obtained when positive values of *A*_*x*_ are considered (OS cases), we compute the mean value of the chemical potential in the three regions (carrier, interface and droplets) for the different cases analyzed. This information is crucial to understand the role played by the energy minimization principle – on which the phase-field method here used to describe the surfactant dynamics is built on – in the transfer rates. Indeed, as the morphology of the dispersed phase is similar among the cases, and thus the effect of the turbulent flow on the transport of surfactant, we can expect that the modulation of the transfer rates can be attributed to the different free-energy levels obtained when *A*_*x*_ is changed. Indeed, if we consider a given concentration of surfactant, the corresponding free energy level will be different in the two phases for nonzero values of the parameter *A*_*x*_ ≠ 0, as shown by eq 5. We can characterize the free energy content in the three region of the multiphase system by computing the average value of the surfactant chemical potential. Differences in the chemical potential will lead to transfer of surfactant from one region to the other (from high chemical potential regions to low chemical potential regions).

Figure 10 shows the time evolution of the average chemical potential in the three regions over time. Left panel refers to WS cases while the right panel to OS cases. The top row shows the results obtained at *We* = 1.5 while the bottom row those obtained at *We* = 3.0. The ES cases results are shown with black curves, as reference. Symbols are used to identify the three regions: carrier (dashed), interface (line), and droplets (dotted) and colors the different values of *A*_*x*_ (same color code as before). Initially, the chemical potential is equal in the three regions as the simulations are initialized with the equilibrium profile for the surfactant concentration. Then, as time evolves, coalescence and breakage phenomena start to take place and the interfacial area decreases (with respect to the initial value) and the average chemical potential in the three regions reach different values. For the WS cases (left column), for both Weber numbers, we observe that the regions characterized by the larger values of the chemical potential is the carrier (dashed lines). This is coherent with the behavior of the transfer rates previously discussed and, as surfactant is depleted from the interface and transferred to the carrier (thus increasing the concentration in the carrier), the corresponding energy level increases. Interestingly, the small of amount of surfactant adsorbed by the dispersed phase (see Figure 9), where surfactant is almost insoluble, only marginally affects the chemical potential value in the dispersed phase (circles) that is only slightly higher than that obtained at the interface (line). Analyzing the effect of the parameter *A*_*x*_, we observe that its influence is minimal. The only noticeable impact appears in the interfacial region and within the droplets, where a decrease of *A*_*x*_ slightly increases the average chemical potential in these regions.

**Figure 10:**
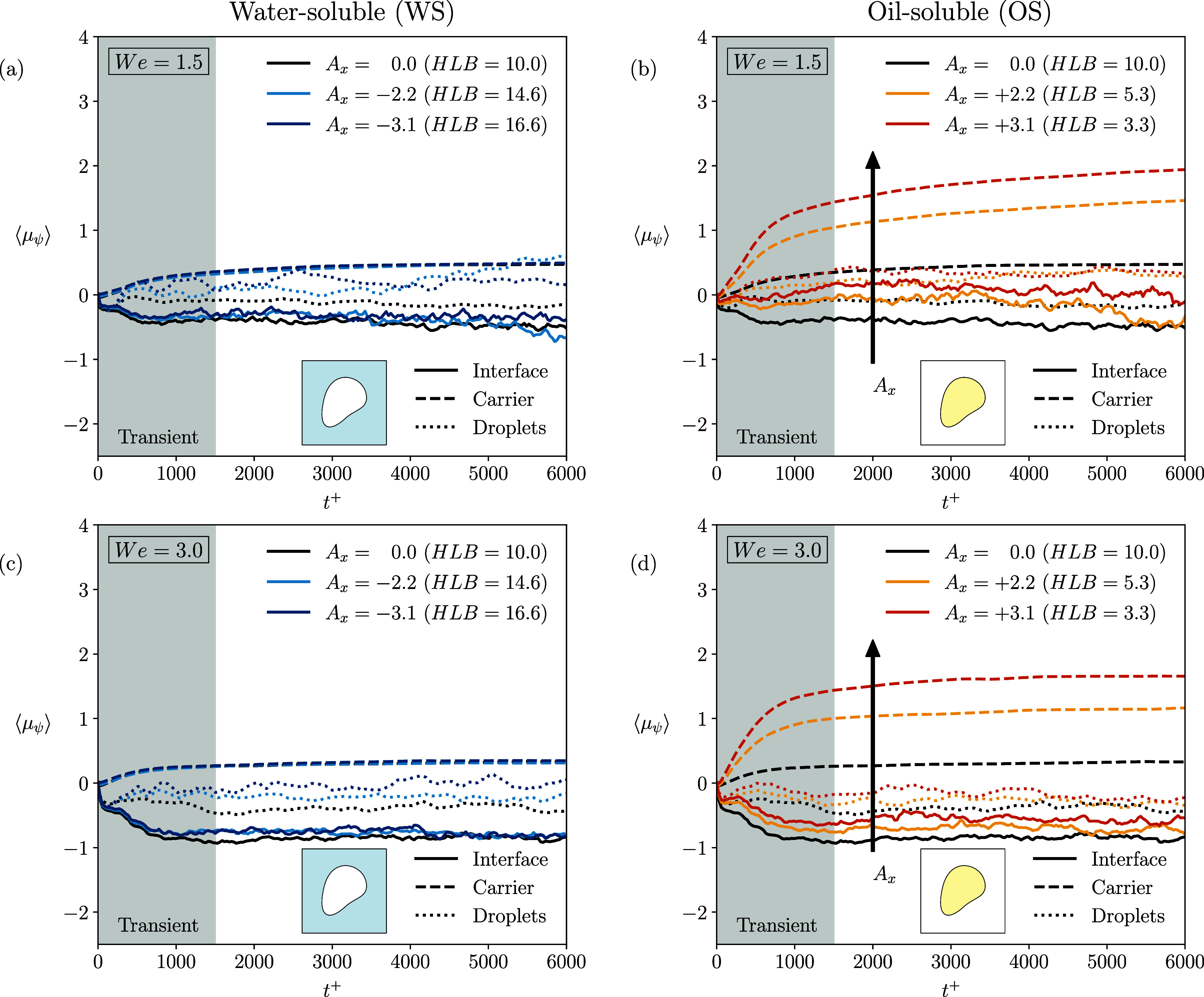
Time evolution of the average chemical potential in the three regions: interface (continuous line), carrier (dashed line), droplets (dotted line). Left column (panels *a* and *c*) shows the WS surfactant cases, while the right column (panels *b* and *d*) refers to the OS surfactant cases. Equally soluble surfactant cases are shown in black in all panels as a reference. Top row refers to *We* = 1.5 while the bottom row to *We* = 3.0. Surfactant molecules will migrate (via diffusion) from high chemical potential regions to low chemical potential regions.

Overall, all WS cases exhibit similar evolution, as the surfactant transferred to the carrier is efficiently absorbed without generating significant energy barriers. Moving to the OS cases (right column), we observe that while the dispersed phase and interface regions are characterized by average chemical potential values similar to those obtained in the WS cases, the carrier phase region exhibits much larger values. This behavior is due to the high energy associated with the presence of surfactant in the carrier phase, where surfactant is almost insoluble. The presence of surfactant in the carrier can be traced back to the external turbulent flow that leads to desorption of surfactant from the interface. However, as a result of the high chemical potential value attained in the carrier, the differences in the chemical potential (more precisely gradients) obtained between the carrier and interface induces a diffusive flux of surfactant from the carrier to the interface and thus produces a modulation of the corresponding transfer rate, as shown in Figure 9b, d. Indeed, we can see that the modulation becomes more evident as the *A*_*x*_ value is increased. The modulation of the transfer rate leads to an increase of the surfactant concentration at the interface, as also observed in Figure 8.

## Conclusions

We examined the transfer dynamics of hydrophilic and lipophilic surfactants in turbulent oil-in-water emulsions, where oil droplets are dispersed in a continuous water phase, and with the surfactant HLB influencing its solubility in each of the two phases. Using a thermodynamically based computational framework, we performed direct numerical simulations to solve the turbulence dynamics and capture the time evolution of both emulsion morphology and surfactant concentration via a PFM. The method is based on two Cahn-Hilliard-like equations–one for the oil/water interface and one for the surfactant–derived from a two-order-parameter Ginzburg-Landau free energy functional, with the surfactant effect on surface tension modeled through an equation of state. Specifically, we investigated the transfer rates of surfactants with varying solubility, distinguishing between water-soluble (high HLB), equally soluble (intermediate HLB), and oil-soluble (low HLB) surfactants. Simulations were carried out in a turbulent channel flow configuration (*Re*_τ_ = 300), where large, deformable surfactant-laden drops were injected. The PFM was modified to account for selective solubility of surfactants in either the water carrier or oil dispersed phase through a skewed term controlled by the asymmetric solubility parameter *A*_*x*_. This parameter allowed us to model three distinct solubility scenarios: water-soluble (high HLB, *A*_*x*_ < 0), equally soluble (intermediate HLB, *A*_*x*_ = 0), and oil-soluble (low HLB, *A*_*x*_ > 0).

We first examined the dispersed phase topology, where no significant changes were observed with respect to surfactant interface concentration. Overall, modifying the surfactant HLB did not notably affect the time evolution of the interface area. However, for the oil-soluble cases, we observed significant differences in the steady-state values of the interfacial area, which varied non-monotonically with HLB. Next, we focused on surfactant dynamics, particularly its distribution at the interface and in the bulk phases. We observed significant differences in interface concentration, with OS lipophilic surfactants exhibiting higher concentrations than WS hydrophilic and ES surfactants. As *A*_*x*_ increased, the surfactant concentration at the interface also increased. To understand the underlying mechanisms, we analyzed surfactant transfer rates between the carrier phase, interfacial layer, and dispersed phase. In the WS cases, the carrier phase efficiently absorbed surfactant depleted from the interface, resulting in a high interface-to-carrier transfer rate. In contrast, for the OS cases, droplets were less effective at adsorbing surfactant, leading to a lower interface-to-droplet transfer rate. Despite this, a significant amount of surfactant was still transferred from the interface to the carrier phase. This counterintuitive result can be attributed to the thermodynamic framework used in the simulations, where surfactant presence in the carrier phase is penalized in the OS cases, leading to a diffusive flux from the carrier p hase back to the interface. This modulation becomes more pronounced as *A*_*x*_ increases, resulting in higher surfactant concentrations at the interface.

We believe that the present results provide valuable insights for emulsion preparation and surfactant selection. Our findings reveal that the selective solubility of surfactants, characterized by their HLB (hydrophilic–lipophilic balance), has a complex effect on adsorption/desorption dynamics, influencing their distribution between the oil and water phases, as well as interfacial concentrations. Although these variations seem to have only a small influence on droplet morphology or interfacial area–suggesting that these properties remain relatively stable within the examined conditions–our findings indicate that careful selection of surfactant HLB–particularly one that favors solubility in the dispersed phase–can enhance interfacial adsorption and, in turn, improve emulsion stability. Building on these insights, future work will investigate emulsion stability more comprehensively, focusing on the interplay between surfactants and small particles trapped at the interface, as well as their combined effects on surface tension.^58^
